# The Perceived Value of Passive Animal Health Surveillance: The Case of Highly Pathogenic Avian Influenza in Vietnam

**DOI:** 10.1111/zph.12212

**Published:** 2015-07-03

**Authors:** A. Delabouglise, N. Antoine‐Moussiaux, T. D. Phan, D. C. Dao, T. T. Nguyen, B. D. Truong, X. N. T. Nguyen, T. D. Vu, K. V. Nguyen, H. T. Le, G. Salem, M. Peyre

**Affiliations:** ^1^ AGIRs‐Animal and Integrated Risk Management Research UnitCIRAD‐French Agricultural Research Center for International DevelopmentMontpellierFrance; ^2^ LADYSS, Enjeux Sanitaires et Territoires, CNRSUniversity Paris‐ouest Nanterre‐La DéfenseNanterreFrance; ^3^ FARAH‐Fundamental and Applied Research for Animals & HealthUniversity of LiègeLiègeBelgium; ^4^ Center for Interdisciplinary Research on Rural DevelopmentVietnam National University of AgricultureHanoiVietnam; ^5^ Faculty of Veterinary MedicineVietnam National University of AgricultureHanoiVietnam; ^6^ National Institute of Veterinary ResearchHanoiVietnam; ^7^ Faculty of Animal Science and Veterinary MedicineNong Lam UniversityHo Chi Minh CityVietnam

**Keywords:** Infectious disease surveillance, economic evaluation, acceptability, participatory epidemiology, stated preference methods, highly pathogenic avian influenza

## Abstract

Economic evaluations are critical for the assessment of the efficiency and sustainability of animal health surveillance systems and the improvement of their efficiency. Methods identifying and quantifying costs and benefits incurred by public and private actors of passive surveillance systems (i.e. actors of veterinary authorities and private actors who may report clinical signs) are needed. This study presents the evaluation of perceived costs and benefits of highly pathogenic avian influenza (HPAI) passive surveillance in Vietnam. Surveys based on participatory epidemiology methods were conducted in three provinces in Vietnam to collect data on costs and benefits resulting from the reporting of HPAI suspicions to veterinary authorities. A quantitative tool based on stated preference methods and participatory techniques was developed and applied to assess the non‐monetary costs and benefits. The study showed that poultry farmers are facing several options regarding the management of HPAI suspicions, besides reporting the following: treatment, sale or destruction of animals. The option of reporting was associated with uncertain outcome and transaction costs. Besides, actors anticipated the release of health information to cause a drop of markets prices. This cost was relevant at all levels, including farmers, veterinary authorities and private actors of the upstream sector (feed, chicks and medicine supply). One benefit associated with passive surveillance was the intervention of public services to clean farms and the environment to limit the disease spread. Private actors of the poultry sector valued information on HPAI suspicions (perceived as a non‐monetary benefit) which was mainly obtained from other private actors and media.

**Impacts**

- Perceived costs and benefits of the passive surveillance system were assessed in three locations of Vietnam. An original study design was applied, which can be qualified as a ‘rapid passive surveillance appraisal’. It combined semi‐structured interviews with visualization tools from participatory epidemiology.
- The perceived value of the passive surveillance system was influenced by different factors including uncertainty in outcomes of reporting, transaction costs, and anticipation of impacts on the poultry market, financial costs supported by local authorities, government support and usefulness of sanitary information.
- Non‐monetary costs and benefits could be quantified by adapting stated preference methods, either contingent valuation or conjoint analysis.

## Introduction

Animal production in developing countries is facing important health issues, with potential consequences for human health (Bonfoh et al., 2010). Adequate allocation of efforts to surveillance and control of animal diseases is becoming even more critical for such countries with limited economic resources. Therefore, the need to optimize the efficiency of animal disease surveillance systems to ensure their sustainability is of primary importance. Passive surveillance, also called reactive surveillance, relies on spontaneous reports of disease suspicions by animal producers, other actors inside or outside the animal production sector (hereafter the private actors of passive surveillance systems) to veterinary authorities, who are locally represented by government veterinarians (hereafter the public actors of surveillance systems) (Hoinville, 2011). It is widely considered as the most cost‐effective way for early detection of outbreaks and to gather information on the disease situation for decision‐making on control strategies (FAO, 2011b). However, in practice, sensitivity and timeliness of passive surveillance are often not optimal, and underreporting of suspicions is considered as a major cause of disease control failure (Drewe et al., 2011; Vergne et al., 2012).

A deep understanding of the decision‐making process for the public and private actors of passive surveillance systems is required to better understand underreporting (Chilonda and Van Huylenbroeck, 2001). Several attempts have been made to analyse this decision‐making process by exploring individuals’ perceptions (Hopp et al., 2007; Elbers et al., 2010; Bronner et al., 2014) and economic, social or cultural constraints impacting their decision (Hickler, 2007; Fearnley, 2011; Sawford et al., 2012; Paul et al., 2013) using anthropological approaches or questionnaire surveys. However, these factors have not yet been integrated within an economic evaluation framework. Indeed, current models of cost–benefit and cost‐effectiveness analysis of passive surveillance systems (Scott et al., 2012) only account for monetary components. To allow for this integration, valuation methods for non‐monetary costs and benefits (i.e. incentives and disincentives that cannot be directly valuated in monetary terms) are to be developed. Participatory epidemiology (PE) proved to be of interest in this respect. This approach is especially aimed at addressing actors’ perception on epidemiologic issues. PE methods are flexible enough to address the wide range of costs and benefits perceived by actors regarding animal health management systems (Catley et al., 2012).

Highly pathogenic avian influenza (HPAI) subtype H5N1 has been present in Vietnam since its first introduction in 2003 (Pfeiffer et al., 2007; Minh et al., 2009). Despite important investments in HPAI surveillance and control programmes, poultry outbreaks are still being reported every year (FAO, 2011a, Department of Animal Health, 2014). The disease is subject to compulsory notification of suspect cases to authorities based on a precise case definition (Department of Animal Health, 2011). The planned official response to these notifications includes the following: investigation of suspect cases followed by laboratory confirmation and culling of confirmed infected flocks, control of bird movements and financial compensations for the owners of the culled flocks (NSCAI, 2012). Underreporting of disease suspicions has been pinpointed as a major limiting factor of the effectiveness of passive surveillance and control programmes against HPAI in Vietnam (Minh et al., 2011). Previous studies have suggested that veterinary authorities are somehow disconnected from the network of private actors of poultry production with regards to animal health (Desvaux and Figuie, 2011). Economic assessments of HPAI control programmes were conducted in Vietnam (Agrifood Consulting International, 2006, Otte et al., 2006; Hong Hanh et al., 2007; Roland‐Holst et al., 2007), but their focus was the direct and indirect financial costs of control measures (culling and restriction on trade), leaving aside costs and benefits especially associated with passive surveillance.

The objective of this study was to identify the perceived costs and benefits of passive surveillance system of HPAI from all actors’ point of view and to propose an innovative way to quantify them.

## Materials and Methods

### Study areas

The study was implemented between 2012 and 2013 in three rural communes which belonged to Hải Dương (HD), Đồng Nai (DN) and Long An (LA) provinces. These three provinces were selected based on their respective characteristics regarding geographical location (HD being located in the north, DN and LA in the south), poultry production and past HPAI reports. Poultry farming represents a significant part of the agricultural systems of these three provinces (General Statistics Office of Vietnam, 2012), and the three provinces are located at short distance from the major urban consumption areas of Hà Nội (HD) and Thành Phố Hồ Chí Minh (TPHCM) (DN and LA). DN had one of the highest concentrations of commercial and industrial poultry production systems in Vietnam, whereas in the two other provinces, such systems were less present. All three provinces reported cases of HPAI H5N1 after the first introduction of the virus in Vietnam in 2003–2005. Despite their location in areas classified as high risk of HPAI (Red River Delta and Mekong River Delta), relatively few cases of HPAI H5N1 had been reported in HD and LA from 2006 to 2011 (2 in HD, 3 in LA). No HPAI case was notified in DN during the same period (Department of Animal Health, 2014). Approvals of the study were obtained from the departments of agriculture and people's committees of the three provinces. Study communes were selected with the help of the provincial veterinary authorities on the basis of their diversity of poultry production systems (presence of small‐, medium‐ and large‐scale farming systems).

### Sampling strategy

The sampling strategy followed a snowball sampling pattern (Sadler et al., 2010). First, several focus group interviews were performed in each study area. Participants were contacted with the help of commune or village authorities. The groups gathered five to twenty poultry farmers. The different production systems present in the study areas were represented, each focus group gathering farmers from the same production system and one or several focus groups being conducted for each production system. Production systems were defined according to the type of production (broiler, layer or parental farms), the species (chicken, duck or quail) and the production scale: small scale or backyard (<100 birds/flock), medium scale (100–1000 birds/flock) and large scale (>1000 birds/flock).

Poultry farmers of each production system who displayed willingness to participate in the study were then asked for individual interviews. The number of these individual interviews was determined by adapting the concept of saturation to the objective of the study (Bowen, 2008): saturation was considered to be reached when 10 additional interviews did not provide any new information on costs and benefits compared with all previous interviews. During this first phase of interviews, other categories of actors were identified as being directly or indirectly impacted by the HPAI passive surveillance process. Individuals belonging to those additional categories of actors and in contact with individuals from the initial sampling frame were then asked to participate in the study. Those who accepted were interviewed individually. Additionally, focus group and individual interviews of government veterinarians were conducted at the village, commune, district and province levels. Finally, a sample of previously interviewed poultry farmers was selected for quantification of the identified non‐monetary costs and benefits of passive surveillance.

### Data collection protocol

Data were collected by teams of two to five researchers and students, including at least one Vietnamese researcher. All of them had a veterinary or animal production degree and did not have any relationship with interviewed participants. All interview team members were previously trained using PE approaches (Mariner and Paskin, 2000). Interviewees were always informed of the purpose of the study and could stop the interview whenever they wanted. Financial indemnities were provided to participants in compensation of the time lost for their normal activities. Relevant amounts of indemnities to provide to participants were evaluated with the help of veterinary authorities: 2.4 USD/interview in HD, and 4.8 USD/interview in LA and DN (conversion rate: 1 U.S. dollar (USD) = 21 000 Vietnam Dong). Names and contact details of interviewees were kept in a secured database only accessible to the research team. All the interviews were conducted in Vietnamese language. Most interviews involved at least one foreign researcher, either as interviewer and/or note‐taker. In such case, one of the Vietnamese researchers directly translated interviewee's responses to him. Questions and responses were directly noted during the interview. Focus group interviews were conducted in official places (commune People's Committee office, district veterinary station, village communal house). Individual interviews were conducted in the private houses or workplaces of participants. In LA, interviews of poultry farmers were all conducted in the commune People's Committee office, as requested by the provincial authorities. Checklists were prepared to keep in mind issues to address during interviews (provided as Figures S1 and S2). Interviews were semi‐structured. General and open questions were used to let the participants focus on what seemed relevant for them, without *a priori* knowledge from the interviewer.

### Assessment of the economic and sanitary context of poultry farming

In focus group interviews of poultry farmers, general information on poultry production systems, value chains, sanitary issues and their management was gathered: (i) actors involved in the poultry value chains (sources of funding and credit, suppliers of feed, breeds and medicines, buyers of farm products) were listed. (ii) Relative importance of general problems affecting poultry farmers and origins of these problems were assessed using simple ranking. (iii) Names used locally for poultry diseases occurring in the area were scored according to their impact on income, rates of mortality and duration using proportional piling (PP) (Catley et al., 2012). Reported names of diseases characterized by both high mortality rate (>50% in one poultry flock) and short duration (<5 days in one flock) were used to define disease suspicions which were referred to in subsequent interviews. (iv) Farmers were then asked which actions were taken when facing a disease suspicion and these actions were scored according to their relative likelihood using PP.

### Qualitative identification of costs and benefits of disease HPAI suspicion reporting

Individually interviewed poultry farmers, government veterinarians and other actors identified by snowball sampling were asked how they perceived costs and benefits associated with disease suspicion reporting using qualitative semi‐structured interviews. (i) They were asked to provide information on the different ways of managing disease suspicion cases when it appeared in poultry farms. (ii) They were asked about the positive and negative consequences of reporting a disease suspicion to authorities. (iii) Impact flow charts were used to identify the negative and positive consequences of disease suspicion reporting for different types of actors. Participants first identified the list of actors impacted by disease suspicion reporting. Then, they assigned different signs and colours to each type of actors to indicate whether the effect was positive or negative (the tool was nicknamed ‘winners–losers list’). Interviewers only used open questions and did not give any suggestions to interviewees. Participants also were asked about their sources of information on disease suspicions, that is the actors they usually obtained information from. With poultry farmers, the sources were simply listed. With other private actors, the sources were scored according to their perceived importance using PP.

### Scoring of relative importance of costs and benefits of disease HPAI suspicion reporting

A subset of backyard, medium‐ and large‐scale broiler chicken farmers of HD study area were asked to score perceived costs and benefits they previously identified according to their impact on their decision to report disease suspicion to veterinary authorities using PP.

### Quantification of non‐monetary costs and benefits using stated preference methods

First, the benefits considered by the individuals when receiving information on disease suspicions were estimated by contingent valuation (Adamowicz et al., 1998). Semi‐structured interviews were performed to list the benefits of early information about the sanitary situation of poultry flocks in the region. The participants had to reflect on how they could use such information and what could be the expected gains or avoided losses from the anticipated actions. Then, contingent valuation was applied. It consisted in offering a virtual contract from a company providing information to the participant at a certain cost. Two factors were considered: the price the participant was willing to pay to receive information in an appropriate timing (i.e. to allow enough time for implementation of prevention and control measures) and the price he was willing to accept as a compensation to deliver information himself within an appropriate timing.

**Figure 1:**
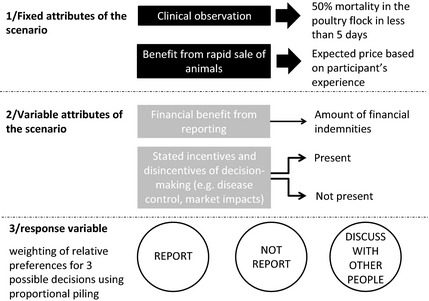
Structure of the conjoint analysis tool applied in the 2012–2013 survey on the perceived value of the HPAI passive surveillance system in Vietnam to quantify non‐monetary costs and benefits associated with disease reporting. Scenarios are composed of fixed attributes and variable attributes. Responses of participants were a scoring of relative preference for three types of decisions using proportional piling.

Second, a modified protocol of conjoint analysis was applied to value the non‐monetary costs and benefits linked to the disease suspicion reporting process (Louviere et al., 2000). The participant had to list and explain the different options he was willing to consider when confronted to a hypothetical scenario of disease suspicion (50% mortality in <2 days) in his chicken flock, and the relative consequences (financial and non‐financial) upon reporting or not reporting the disease suspicion to the authorities. Then, he was asked to ascribe a relative likelihood to the three possible actions: (i) reporting the disease suspicion to authorities, (ii) not reporting the disease suspicion to authorities, and (iii) discussing with other people in the community about the need to report or not (Fig. 1). The objective of the third option was to give a possibility for the participant to opt out, as well as a possibility for him to give more detailed explanation of the social interactions along the decision‐making process. The likelihood of each option was quantified using PP. Different scenarios were then tested by varying the levels of indemnities provided by the government upon report. The motives for the stated likelihoods were assessed at each step and considered as incentives or disincentives of the decision‐making. The participant was then asked to assign likelihoods to each action in situations where the incentives and disincentives considered were not applicable (e.g. assign likelihood of each action when considering that authorities provide or do not provide help in disease management following a disease suspicion report). According to the conjoint analysis framework, the presence and absence of these incentives and disincentives were considered as the attributes to be valued through the different choice scenarios (Fig. 1).

The contingent valuation tool to assess the benefits from receiving disease suspicion information was progressively built in six pilot interviews with broiler chicken producers of HD. Then, it was tested on 21 broiler chicken producers in HD. The conjoint analysis tool was progressively built in six pilot interviews with broiler chicken producers of HD province. Then, it was tested on 17 broiler chicken producers of HD province and six broiler chicken producers of DN.

### Data analysis

Qualitative data were analysed using thematic analysis (Graneheim and Lundman, 2004). Meaning units, that is information or judgments expressed in interviews, were attributed specific codes. Codes were then grouped into subthemes and themes. Identified themes corresponded to specific factors influencing the perception of the HPAI passive surveillance system by participants, either positively or negatively. Each subtheme and theme was linked to the number of interviews it was extracted from. Moreover, to be considered as relevant, themes and subthemes that concerned several categories of actors had to be mentioned by participants from all the concerned categories.

All statistical analyses were made using R.2.15.3 software (R Core Team, 2014). Results from contingent valuations were analysed through descriptive statistics. Degree of agreement between interviewees and groups of interviewees’ rankings and scores obtained by PP was assessed by nonparametric Kendall test of concordance (Legendre, 2005), using kendall.global function of vegan package (Oksanen et al., 2014). Statistical significance of the Kendall coefficient was shown by permutation test.

Results from the adapted conjoint analysis method were analysed considering the stated likelihoods of action as probabilities of choice. Being collected following distinct interview processes according to each individual case, data were analysed individually. To allow for the statistical estimation of utility coefficients of the different scenario attributes with standard statistical package, each individual probability gathered through PP was simulated as resulting from a mock sample (*n* = 100 for the each scenario). A multinomial logistic regression model was applied to derive the monetary values of attributes (Louviere et al., 2000) from data collected for each individual, using the mlogit package (Croissant, 2013):

$$\text{Pr}=\frac{\text{exp}\;(brXr)}{\sum \text{exp}\;(bjXj)},$$

with Pr being the probability of the individual choosing the report option, **X** being the vector of the attributes of the scenario (non‐monetary and monetary incentives and disincentives), **b** being a vector of utility coefficients of the scenario attributes to be estimated by the model, r being the report option and j being the choice set.

## Results

### Study sample size

Nineteen focus group interviews of farmers were carried out, including a total of 189 farmers and covering eight production sectors (chicken: 4, duck: 3, quail: 1) (Table 1). Furthermore, one focus group interview with government veterinarians was conducted in each study area, including a total of 35 individuals. Individual interviews were carried out between 1 and 3 times with 80 farmers, 54 private actors and 13 government veterinarians (Table 2).

**Table 1:** Overview of the number of focus group interviews (*n* = 22) conducted per province and production sector

Type of actor	Specie	Sector	Study area		
			HD	DN	LA
Poultry farmers	Chicken	Backyard	4	2	0
		Medium‐scale broiler	2	0	1
		Large‐scale broiler	1	2	2
		Medium‐scale parental	0	0	1
	Duck	Medium‐scale broiler	0	0	1
		Large‐scale broiler	0	1	0
		Medium‐scale parental	0	0	1
	Quail	Large‐scale layer	0	1	0
	Subtotal		7	6	6
Government veterinarians			1	1	1
Total			8	7	7

**Table 2:** Overview of the number of individual interviews (*n* = 149) conducted per province and type of actor

Type of actor	Species	Sector	Study area			Other
			HD	DN	LA	
Poultry farmer	Chicken	Backyard	6 (3)^a^	7	0	
		Medium‐scale broiler	22 (7)^a^	0	3	
		Large‐scale broiler	12 (4)^a^	7	4	
		Medium‐scale parental	0	0	3	
	Duck	Medium‐scale broiler	0	0	3	
		Large‐scale broiler	0	5	0	
		Medium‐scale parental	0	0	4	
	Quail	Large‐scale layer	0	4	0	
	Subtotal		40	23	17	
Other private actors	Medicine sellers		3	4	5	
	Feed sellers		6	2	4	
	Chicken trader–slaughterer		6	3	4	
	Veterinary technician of feed or pharmaceutical company		8	6	0	
	Journalist	National newspaper	0	0	0	4
		Local newspaper	0	1	0	
	Subtotal		23	16	13	4
Government veterinarians			5	5	3	
Total			68	44	33	4

a In brackets: number of farmers who were also asked to score the relative importance of costs and benefits.

Several private actors selected by snowball sampling refused to participate in the study: feed sellers (HD: 0, DN: 3, LA: 0), medicine sellers (HD: 1, DN: 1, LA: 0), poultry traders–slaughterers (HD: 2, DN: 3, LA: 0) and company technicians (HD: 0, DN: 4). Stated reasons for refusing were lack of time availability. Government veterinarians were present during three focus group interviews of poultry farmers in DN and all focus group interviews in LA. In LA, they were also present during seven of 17 individual interviews of poultry farmers, three of five interviews of medicine sellers, two of four interviews of feed sellers and two of four interviews of poultry traders.

### General problems affecting poultry farmers

Rankings of problems mentioned by poultry farmers in focus group interviews are presented in Table 3. Output price instability was mentioned as the most important problem in most focus group interviews. Other first or second ranking problems were poultry diseases and increase in industrial feed prices. Kendall coefficient of concordance between rankings made in different groups was equal to 0.68. Kendall coefficients of concordance between groups of farmers from similar production scales (medium‐ and large‐scale farms) were equal to 0.82 and 0.62, respectively, which implied a high degree of agreement between group discussions. Both coefficients were statistically significant (*P* < 0.01). Causes of output price instability mentioned by groups of farmers were imports of poultry products from abroad (HD, DN, LA), normal seasonality of price associated with traditional festivals (HD, DN, LA), HPAI notifications (DN, LA) and speculation of agro‐industrial companies (DN).

**Table 3:** General problems reported in focus group interviews of poultry farmers involved in the 2012–2013 survey on the perceived value of the HPAI passive surveillance system in Vietnam, ranked according to their relative perceived importance

Farming scale	Study area^a^	Type of production	Type of problem^b^										*W* c
			PD	HW	LC	PI	CP	FP	PVP	CQ	LTL	WV	
Large(>1000 birds/flock)	HD	Broiler chicken	5	2	3	4	0	1	0	0	0	0	0.62
	LA	Broiler chicken	0	0	1	3	0	2	0	0	0	0	
	DN	Broiler chicken	0	0	0	1	0	0	0	0	0	0	
		Broiler chicken	2	0	0	4	0	3	0	0	0	1	
		Broiler duck	3	0	0	4		2	0	1	0	0	
		Layer quail	2	0	0	3	1	0	0	0	0	0	
Medium (100–1000 birds/flock)	HD	Broiler chicken	6	0	5	7	2	8	1	4	3	0	0.82
	LA	Broiler chicken	1	0	2	4	0	3	0	0	0	0	
		Broiler duck	1	0	0	3	0	2	0	0	0	0	
		Parental chicken	2	0	0	3	0	1	0	0	0	0	
		Parental duck	1	0	0	4	0	3	0	0	0	2	
Small (<100 birds/flock)	DN	Backyard chicken	2	0	0	3	0	0	0	0	0	1	

The higher the rank, the more important the problem is perceived. Colour code: dark grey: most important mentioned problem; light grey: second most important mentioned problem.

a DN, Đồng Nai; HD, Hải Dương; LA, Long An.

b PD, Poultry diseases; HW, high wages of workers; LC, limited capital; PI, output price instability; CP, chick/duckling price; FP, increasing feed price; PVP, increasing price of veterinary products; CQ, chick/duckling quality; SG, stunted growth; LTL, limited technical level; WV, weather variation.

c W: Kendall coefficient of concordance between rankings of groups of similar farm scales.

### Management of suspicion diseases by farmers

Among poultry diseases mentioned in focus group interviews with farmers, several ones potentially matched the HPAI suspicion definition, that is caused more than 50% mortality in poultry flocks in <5 days, including Newcastle disease, fowl cholera, Gumboro disease and duck plague. Scores of the different options considered by poultry farmers according to their likelihood are presented in Table 4. Scores could not be collected in focus groups in LA. Poultry farmers of this study area displayed hesitation to do the exercise in the presence of government veterinarians. Differences of responses were observed between focus group interviews. The Kendall coefficient of concordance was equal to 0.34, meaning limited agreement between groups (*P* < 0.01). Kendall coefficients in the different classes of farm scales were 0.43 (large scale), 0.87 (medium scale) and 0.44 (backyard), with limited statistical significance (respective p values were 0.10, 0.10 and 0.03). Kendall coefficients in the different study areas were 0.68 (HD) and 0.58 (DN), and both were statistically significant (*P* < 0.01). There was a higher agreement between groups of interviewees of similar study areas than between groups of similar farming scales. The main mentioned options were asking support from a private actor (feed seller, veterinary shop, feed company), rapid sale of the animals, warning of other farmers and self‐reliance. Reliance on veterinary shops was mentioned in HD and DN. Rapid sale and reliance on feed sellers were mainly mentioned in HD. Reliance on an agro‐industrial company was the highest scoring option of large‐scale broiler duck farmers of DN because of the common contract agreements linking them with a company. Quail farmers gave the highest score to self‐reliance due to the lack of availability of private or public veterinarians trained in quail treatment.

**Table 4:** Scores attributed by proportional piling to relative likelihoods of decisions operated when facing a disease causing high mortality (>50% of affected flocks) in a short time period (<5 days) in focus group interviews of poultry farmers involved in the 2012–2013 survey on the perceived value of the HPAI surveillance system in Vietnam

Production scale	Production system	Study area^a^	Relative likelihood of decision^b^							*W* c
			PF	RS	FS	VS	AIC	VA	SR	
Large (>1000 birds/flock)	Broiler chicken	HD	17	28	18	37	0	0	0	0.43
	Broiler chicken	DN	0	0	0	100	0	0	0	
	Broiler duck		9	0	0	24	67	0	0	
	Layer quail		0	0	0	30	6	0	64	
Medium (100–1000 birds/flock)	Broiler chicken (*n* = 2)	HD	39	0	36	18	7	0	0	0.87
			38	19	27	16	0	0	0	
Small (<100 birds/flock)	Backyard chicken (*n* = 4)	HD	17	25	49	9	0	0	0	0.44
			27	0	56	17	0	0	0	
			8	62	25	5	0	0	0	
			6	7	53	26	0	8	0	
	Backyard chicken (*n* = 2)	DN	0	0	0	80	0	0	20	
			31	0	0	69	0	0	0	

Colour code: dark grey: most likely; light grey: second most likely.

a The study areas: HD, Hải Dương; DN, Đồng Nai; LA, Long An.

b Decision: PF: warning of other poultry farmers; RS: rapid sale of animals; FS: seeking support from a feed seller; VS: seeking support from a veterinary shop; AIC: seeking support from an agro‐industrial company; VA: report to veterinary authorities; SR: self‐reliance.

c W: Kendall coefficient of concordance between scorings of groups of similar farm scales.

### Qualitative identification of factors influencing the perceived value of passive surveillance: identified themes

Six themes related to factors influencing the perceived value of avian disease passive surveillance were identified from individual interviews. Related non‐monetary costs and benefits associated with these themes are summarized in Table 5.

**Table 5:** Non‐monetary factors influencing the perceived value of HPAI passive surveillance system identified in individual semi‐structured interviews of the 2012–2013 survey on HPAI surveillance in Vietnam

Effect	Type	Explanation	Study area^a^	Number of actors mentioning it in individual interviews^b^					
				PF	GV	FS	MS	PT	CT
Negative	Uncertainty in the outcomes of reporting	Uncertainty of intervention/support of upper level authorities	HD	**24**	**6**	**3**	0	0	0
			DN	**9**	0	**1**	**2**	0	**1**
			LA	**2**	0	0	0	0	
	Transaction costs	Time before intervention and indemnification, administrative fees and procedures, distance	HD	**13**	0	**3**	**1**	**1**	0
			DN	**7**	0	0	**1**	0	**1**
			LA	**1**	0	0	0	0	
	Limits of local government resources	Pressure for limiting local governments’ expenditures in disease control measures	HD	**2**	**5**	0	0	0	0
			DN	**1**	0	0	**1**	0	**1**
			LA	0	0	0	0	0	
	Market impact	Poultry price fluctuation due to rapid sale, reduced demand, pressure of traders, movement restrictions	HD	**27**	**4**	**5**	**1**	**6**	**5**
			DN	**19**	**3**	**1**	**2**	**3**	**5**
			LA	**17**	**1**	**2**	**5**	**4**	
Positive	Disease management	Cleaning/disinfection of farms and of the environment	HD	**20**	**3**	**1**	0	0	0
			DN	0	**3**	0	0	0	0
			LA	**6**	**2**	0	0	0	
	Usefulness of information	Information on disease outbreak occurrence: help in disease prevention and anticipation of market impact	HD	**22**	c	**5**	**2**	c	**5**
			DN	**12**	c	**1**	**4**	c	**6**
			LA	**15**	c	**3**	**4**	c	

a HD, Hải Dương; DN, Đồng Nai; LA, Long An.

b PF, poultry farmer; GV, government veterinarian; FS, feed seller; MS, medicine seller; PT, poultry trader; CT, company technician.

c The question of the utility of information was not discussed with government veterinarians and poultry traders.

Positive values are bold and shaded.

#### Theme 1. Reporting disease suspicion: a choice under uncertainty

**Figure 2:**
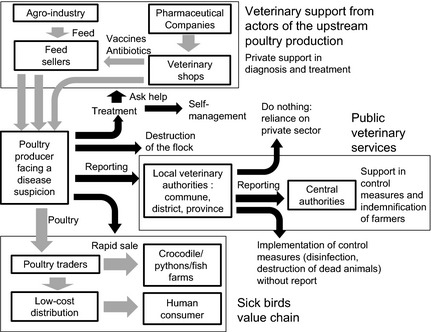
Mentioned choices operated by poultry farmers and government veterinarians interviewed during the 2012–2013 survey on the perceived value of the HPAI passive surveillance system in Vietnam when facing disease suspicion (grey arrow: commercial linkage, black arrow: decision).

Four types of options for disease management were considered by poultry farmers (Fig. 2): (i) solving the disease problem through diagnosis and treatment (which could require the intervention of private actors, including medicine sellers, feed sellers, technicians of the agro‐industrial or pharmaceutical industry), (ii) destruction of animals (through burying, burning or disposal in the environment), (iii) reporting to the local government veterinarian or (iv) sale of animals. Trade of sick or dead animals was mentioned by poultry farmers (HD: *n* = 25, DN: *n* = 15, LA: *n* = 7) and poultry traders (HD: *n* = 6, DN: *n* = 3, LA: *n* = 1). In HD, broiler chicken farmers indicated that they were able to sell sick broiler chickens above 1 kg at 33.3–83.3% of the market price (*n* = 17). In DN, broiler farmers indicated that sick and dead animals could be sold to farms rearing pythons or crocodiles at 10–20% of the market price (*n* = 10). In both study areas, they mentioned that sick birds were slaughtered and prepared for human consumption (HD: *n* = 8, DN: *n* = 4). Poultry farmers’ perceived likelihood of treatment efficacy and effectiveness of support from the private veterinary actors influenced their likelihood of attempting diagnosis and treatment (HD: *n* = 12, DN: *n* = 10, LA: *n* = 10). The option of selling animals was influenced by the anticipation of the sale price by the farmers. This price would be defined by the age, degree of sickness known by the trader and the overall market price level (HD: *n* = 25, DN: *n* = 6). Confronted with disease suspicion, owners of broiler flocks in the early stage of growth would not consider rapid sale, whereas owners of fully grown animals could directly contact the trader. The economic benefit expected by poultry farmers from reporting to veterinary authorities was the culling of birds associated with financial indemnities (HD: *n* = 37, DN: *n* = 20, LA: *n* = 17). The perceived likelihood of the reaction of veterinary authorities following reporting of HPAI suspicion (i.e. whether veterinary authorities were expected to intervene or not) was therefore an important factor considered by poultry farmers in their reporting decision (HD: *n* = 24, DN: *n* = 9, LA: *n* = 1). High or rapid poultry mortality could be associated with several potential diseases, whereas only HPAI was perceived as notifiable by poultry farmers. The negative perception from poultry farmers of the authorities’ response was also linked to the lack of trust in government veterinarians’ competence and willingness to help them (HD: *n* = 10, DN: *n* = 12, LA: *n* = 3).

#### Theme 2. Transaction costs related to reporting

Poultry farmers reported transaction costs associated with disease suspicion reporting to veterinary authorities (HD: *n* = 13, DN: *n* = 7, LA: *n* = 1).

Reported delays in getting financial indemnities varied between several months and more than one year, which was perceived as too long by the farmers. Semi‐commercial and commercial poultry producers bought their feed (all study sites) and/or the veterinary products (LA) on credit. They faced short payback periods (commonly one production cycle of 2–4 months) and incurred increased interest rates and/or threats by their creditors in case of late refunding. This pressure on debt was confirmed by both poultry farmers (HD: *n* = 7, DN: *n* = 4, LA: *n* = 6) and feed sellers (HD: *n* = 3, DN: *n* = 1, LA: *n* = 3). Therefore, farmers expressed a relatively high preference for getting money quickly, that is by selling the animals.

Besides, cumbersome and time‐consuming procedures were associated with the reporting option by poultry farmers in HD (*n* = 5). Additional transaction costs were also associated with the involvement of veterinary authorities (HD: *n* = 3, DN: *n* = 4, LA: *n* = 1). Poultry farmers perceived government veterinarians provided better support (in terms of financial indemnities and disease control) to their own relatives, and that additional fees could be required from poultry farmers to officially report the disease suspicion or to provide compensations and disinfectants.

#### Theme 3. Limits of local authorities resources

Government veterinarians associated reporting with an obligation of expenditure in disease control. According to government veterinarians (HD: *n* = 5, DN: *n* = 5, LA: *n* = 3), in case of notification, disease control costs, in terms of equipment, consumables and labour, were supported by province government (HD) or district government (DN) or were shared between the province, district and commune governments (LA). An additional help was provided by the central government in case the local government ran out of fund. Government veterinarians interviewed individually in HD (*n* = 4) expressed concerns about sparing the local government's financial resources. They suggested that this problem could be resolved by informally helping poultry farmers managing disease cases without making official reports.

#### Theme 4. Anticipation of market impacts

**Figure 3:**
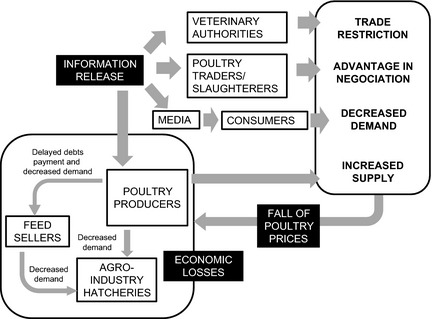
Market effects of the release of information on HPAI suspicions perceived by participants of the 2012–2013 survey on the perceived value of the HPAI passive surveillance system in Vietnam.

Poultry farmers anticipated that the release of information on disease suspicions would cause a dramatic drop of poultry market prices (HD: *n* = 27, DN: *n* = 19, LA: *n* = 17). Four explanatory factors were given (Fig. 3). First, poultry farmers informed would massively sell their flocks earlier in an attempt to avoid the infection or the implementation of control measures (HD: *n* = 8, DN: *n* = 4, LA: *n* = 15). Second, poultry consumers adapted their purchase in response to HPAI suspicion information, switching from poultry products to substitute goods such as pig products (HD: *n* = 11, DN: *n* = 15, LA: *n* = 17). Third, the poultry traders used the released information as a bargaining advantage to lower the purchase price. This was confirmed by poultry farmers (HD: *n* = 19, DN: *n* = 8, LA: *n* = 9) and poultry traders (HD: *n* = 4, DN: *n* = 1, LA: *n* = 3). Last, poultry movement restrictions limited the commercialization of farm products (HD: *n* = 20, DN: *n* = 9, LA: *n* = 14). Movement restrictions were used by traders as an additional justification to decrease the poultry purchase price. Besides the benefits for traders, participants reported several negative consequences of market impacts on actors of the upstream sector (the feed sellers, hatcheries and the agro‐industry) who were not involved in the reporting process (Table 6). These actors faced a decreased demand of inputs from poultry farmers affected by price falls and delays in the payment of their credits. This information was obtained from poultry farmers (HD: *n* = 14, DN: *n* = 4, LA: *n* = 9), feed sellers (HD: *n* = 3, DN: *n* = 1, LA: *n* = 1), medicine sellers (HD: *n* = 1, DN: *n* = 1, LA: *n* = 3) and company technicians (HD: *n* = 5, DN: *n* = 3).

**Table 6:** Reported perceived negative and positive impacts on different types of actors associated with disease suspicion reporting to the veterinary authorities in Vietnam during the 2012–2013 survey on the perceived value of HPAI passive surveillance

Category of actor	Type	Explanation
Unaffected poultry producers	Disadvantage	Loss of revenue caused by decreased commercial value of poultry
Feed sellers	Disadvantage	Loss of revenue due to delays in debt payments and decreased purchase by farmers who incurred revenue losses
Hatcheries	Disadvantage	Loss of revenue due to decreased purchase by farmers who incurred revenue losses
Medicine sellers	Both	(−) Loss of revenue due to delays in debt payments (+) Benefits due to higher sales of medicines to farmers for disease prevention
Agro‐industry	Disadvantage	Loss of revenue due to decreased purchase by famers who incurred revenue losses
Poultry traders/slaughterers	Advantage	Increased profit margin through purchasing poultry at lower price and selling it at the normal market price
Poultry consumers	Both	(−) Fear of infected products (+) Possibility to avoid potentially infected products or to buy it at cheap price

In southern Vietnam, poultry farmers explicitly referred to recent cases of HPAI notifications in provinces of the Mekong River Delta, which were followed by a drop in prices in other unaffected provinces. The reported drop in chicken prices indicated by poultry farmers ranged from 13 to 35% in DN (*n* = 4) and from 21 to 50% in LA (*n* = 32), while the drop in duck prices ranged from 23 to 25% in DN (*n* = 2) and from 35 to 48% in LA (*n* = 14). These drops were always followed by a period of higher prices caused by a deficit in poultry supplies. Poultry farmers of HD did not mention drops of prices specifically related to HPAI announcements. Rather, the latter mentioned cases when diseases matching the HPAI case definition affected the local sale price when the information was spread, with or without notification (HD: *n* = 8). Owners of large‐scale commercial farms considered they incurred higher costs than smaller scale farmers (HD: *n* = 5, DN: *n* = 4). Such farmers sold a larger part of their production in distant areas or cities and therefore were more dependent towards intermediate actors and more vulnerable to market changes.

Protecting commercial interests of poultry producers was a concern of local authorities, and fear of market impacts could influence their decision (HD: *n* = 2, DN: *n* = 3). In DN, anticipation of market impacts by local authorities was mentioned by poultry farmers as the main cause of absence of disease notifications (*n* = 13).

#### Theme 5. The benefits associated with government intervention in disease management

In HD and LA, support by veterinary authorities in disease management was considered a benefit by poultry farmers (HD: *n* = 20, LA: *n* = 6). Given reasons were clearance of the farms from the pathogen, avoidance of disease spread to other farms, avoidance of environmental pollution and protection of public health. Environmental pollution and associated sanitary risks and discomfort of villagers due to the release of dead animals in the rice fields, water ponds and rivers when disease happened was perceived as a concern by poultry farmers (HD: *n* = 11, LA: *n* = 3). In HD, environmental pollution was specifically mentioned as a motive for reporting by poultry farmers (*n* = 6) and government veterinarians (*n* = 1). Protection of public health also was considered a reason for reporting disease suspicion (*n* = 10). Benefit of government intervention in disease management was not mentioned in DN.

#### Theme 6. The benefits of receiving information

Poultry farmers also perceived receiving information about disease cases as a benefit (HD: *n* = 21, DN: *n* = 13, LA: *n* = 16). Indeed, they used this information to implement prevention measures. The main preventive measures mentioned were disinfection (HD: *n* = 6, DN: *n* = 6, LA: *n* = 7) and vaccination (HD: *n* = 9, DN: *n* = 6, LA: *n* = 3).

Information on disease suspicions could also result in early sale of adult animals (HD: *n* = 7, DN: *n* = 3, LA: *n* = 15) in anticipation of the disease spread and drop in prices.

Speculation on price evolution was mentioned by poultry farmers in HD, that is stocking young animals to sell them at the end of the epizootic, awaiting the rise in prices due to the shortage of poultry on markets (HD: *n* = 9). Actors of the upstream sector (feed sellers, medicine sellers, company technicians) benefited from information on disease suspicions as it enabled them to anticipate variations of market prices and to warn their customers (HD: *n* = 14, DN: *n* = 13, LA: *n* = 11).

Poultry farmers who were interrogated on the sources of poultry health information they used (HD: *n* = 17, DN: *n* = 15, LA: *n* = 15) mentioned the following sources: other poultry farmers (HD: *n* = 11, DN: *n* = 12, LA: *n* = 9), veterinary shops (HD: *n* = 5, DN: *n* = 7, LA: *n* = 7), media (TV, newspaper, radio) (HD: *n* = 4, DN: *n* = 8, LA: *n* = 15), agro‐industrial or pharmaceutical industry (HD: *n* = 5, DN: *n* = 5, LA: *n* = 1), veterinary authorities (HD: *n* = 3, DN: *n* = 0, LA: *n* = 12), public loudspeakers (HD: *n* = 0, DN: *n* = 2, LA: *n* = 6), feed sellers (HD: *n* = 2, DN: *n* = 1, LA: *n* = 1) and poultry traders (HD: *n* = 0, DN: *n* = 1, LA: *n* = 1).

Scores of sources of information mentioned by other private actors (feed sellers, medicine sellers, technicians of agro‐industrial companies) are presented in Table 7. For feed sellers, the main mentioned sources were poultry farmers, veterinary shops and agro‐industrial companies. For veterinary shops, main mentioned sources were poultry farmers, pharmaceutical companies, veterinary authorities and the media. For technicians of agro‐industrial companies, main mentioned sources were poultry farmers, feed sellers and other technicians. Values of the Kendall coefficient of concordance applied on scores of information sources were high for all types of private actors (feed sellers: *W* = 0.53, veterinary shops: *W* = 0.60, technicians of agro‐industrial companies: *W* = 0.71). All coefficients were statistically significant (*P* < 0.01). Journalists interviewed mentioned that media gathered information from private informants at the local level and from official notifications of authorities (*n* = 4). Besides, according to journalists (*n* = 4), government veterinarians (DN: *n* = 2) and poultry farmers (HD: *n* = 12), disease suspicions could be advertised by the media before notification of the authorities.

**Table 7:** Scores attributed to each source of information on poultry disease suspicions by interviewed upstream private actors of animal disease management in the 2012–2013 survey on the perceived value of the HPAI surveillance system in Vietnam

Private actor	Feed sellers	Veterinary shops	Technicians of agro‐industrial companies
*n*	9	10	8
Sources			
PF^a^	40 (10–64)	39 (24–62)	24 (11–49)
VS^a^	17 (0–25)	6.5 (0–22)	0 (0–5)
FS^a^	0 (0–21)	0 (0–1)	21.5 (0–31)
BS^a^	0 (0–7)	0 (0–12)	2.5 (0–7)
PT^a^	4 (0–27)	0 (0–18)	0 (0–11)
AIC^a^	15.5 (0–45)	0 (0–19)	35 (30–51)
PC^a^	0 (0–8)	17.5 (0–37)	6 (0–16)
P^a^	0 (0–0)	0 (0–15)	0 (0–0)
VA^a^	0 (0–14)	14.5 (0–28)	0 (0–6)
LS^a^	0 (0–20)	0 (0–0)	0 (0–0)
M^a^	8 (0–20)	14.5 (0–29)	3 (0–43)
VH^a^	0 (0–4)	0 (0–0)	0 (0–0)
*W* b	0.53	0.60	0.71

Presentation of scores: median (minimum–maximum).

a PF, poultry farmers; VS, veterinary shops; FS, feed sellers; BS, breed suppliers/hatcheries; PT, poultry traders; AIC, agro‐industrial companies; PC, pharmaceutical companies; P, people in general; VA, veterinary authorities; LS, loudspeakers; M, media; VH, village heads.

b W: value of Kendall coefficient of agreement in each of the three classes of actors.

### Scoring of the weight of identified costs and benefits in farmers’ decision‐making

Fourteen broiler chicken farmers of HD, with flock sizes ranging from 100 to 7000 animals, were asked to score the weight of costs and benefits they previously mentioned on their reporting decision‐making using PP. Median score attributed to personal financial interests (influenced by transaction costs, uncertainties of access to financial indemnities and possibility so sell sick animals) was 37.5 (range: 10–65), median score given to disease management was 32 (range: 12–79) and median score given to market impacts was 28 (range: 0–46). The value of Kendall coefficient of concordance was 0.66, indicating a high degree of agreement between responses (*P* < 0.01).

### Quantification of non‐monetary costs and benefits

The relevant disincentives of poultry farmers for reporting were the fear of being responsible for the losses incurred by other producers and feed sellers in case of notification, and the transaction costs. From the 17 interviews performed in HD with broiler chicken producers, 11 results were interpretable. Failures to obtain interpretable results arose from inability or unwillingness of participants to envisage hypothetic scenarios. For five farmers, the effect on prices and resulting losses for other farms did not affect their decision (null cost), and for five other farmers, this cost had an impact that could be quantified; the median value was 442 USD (range: 108–2979 USD) (exchange rate: 1 USD = 21 000 Vietnam Dong). One farmer said the impact on price was absolutely intolerable. Five farmers did not mention the transaction costs of reporting. For six other farmers, these transaction costs could be estimated as a median value of 694 USD (range: 236–1081 USD). Seven farmers did not mention the benefit of help in disease management. For four other farmers, this benefit could be estimated as a median value of 292 USD (range: 248–829 USD). Six pilot interviews were implemented in DN and did not provide interpretable results, as participants stated the given scenarios were too dissociated from reality. According to them, poultry disease management was generally under the control of the private sector and they lacked historical records of disease notification in poultry (no poultry diseases had been notified since 2005).

A quantified value of acceptable price for getting information on disease suspicion (willingness to pay) could be obtained from 13 of the 21 interviews performed in HD with broiler chicken producers. The median value was 0.04 USD (range: 0.005–0.05 USD) per chicken per cycle, which corresponds to about 1% of the chicken sale price.

## Discussion

### Economic impact of HPAI passive surveillance in Vietnam

At poultry farmers’ level, risk aversion, time preference, lack of trust in veterinary services and compensation policy were key components of their decision‐making process. Although the level of compensation might be close to the poultry market price, rapid sale might still be perceived as a quicker and safer alternative to reduce income losses.

The study also demonstrated that the choice of not releasing poultry health information to avoid market disturbance was a major feature of the decision‐making process of several types of actors, at different scales of operation, including veterinary authorities. Output price instability was ranked as a higher concern than diseases in most farmers’ focus group interviews, and HPAI notification was pointed as one cause of this instability in the two southern study areas. This result further underlines the importance given to market impacts in farmers’ decision‐making. Such market disturbances have been well characterized and quantified for avian influenza in several countries by multimarket or even computable general equilibrium models (Rodriguez et al., 2007; Diao, 2009; Thurlow, 2011). These impacts are complex and entail many distributional effects, besides the overall loss for society. Some examples may be extracted from the present study. First, consumers may transfer their demand for meat from poultry to swine products, the latter sector then generating more profit. Second, from their use of health information, traders also generate more profit during epizootics at the expense of poultry producers. Third, some poultry farmers adopt alternative strategies such as timing the sale of their flocks in the period of high deficit of poultry supply that just follows the epizootics to generate higher profits. Effects of sharing disease suspicion information on poultry prices may vary from one area to the other, depending on the scale of production (large‐scale farms being more impacted), the proximity to consumers and the influence of intermediate actors on price. Previous consumers’ surveys in Vietnam showed that fears of HPAI risk for human caused a momentary reduction of purchase of poultry products but, on the long run, did not significantly impact consumption habits (Figuie and Fournier, 2008; Figuie et al., 2013). Consumption patterns and demand for food safety vary between the urban areas of Hanoi and Ho Chi Minh City (Soares Magalhaes et al., 2007; Ifft et al., 2010a) although in both areas HPAI was ranked first in consumers’ concerns regarding poultry products’ safety (Ifft et al., 2010a). Consideration given by local authorities to economic impacts of disease notification on farmers also depended on the contribution of poultry production to their area's overall income.

Private actors of the poultry production expressed a need for early information on occurrences of poultry diseases. So far, the public passive surveillance system in Vietnam is not adapted to this requirement and hence met by the private information networks. A major part of the Vietnamese poultry production is concentrated in small‐scale farming systems, and most farmers cannot afford constant investments in biosecurity and prevention measures. Information on disease occurrences is especially useful for such farmers who can adapt their choices (preventive measures or early sale of animals) according to the obtained information on sanitary threats.

The role of the media as ‘enhancer’ of public passive surveillance was highlighted. The media collected information outside the institutional networks. It communicated this information in response to a need of diverse actors to get awareness of the epizootic situation including public passive surveillance stakeholders. These observations confirm the positive influence that informal disease surveillance means can exert on disease suspicion reporting (Davies, 2012). Nevertheless, substitution of veterinary authorities by media in the supply of poultry health information (some HPAI suspicion cases being announced by media before their official notification) also contributed to the distrust of private actors towards the public surveillance system.

Our results did not confirm that passive surveillance of small‐scale poultry farms is less effective than passive surveillance in larger commercial productions. According to previous studies, private large‐scale farmers depend more on itinerant traders and distant large markets to sell their products, while small‐scale farmers tend more to sell their products locally and directly to consumers (Tung and Costales, 2007; Fournie et al., 2012). Therefore, large‐scale farmers might be more vulnerable to drops of price arising from asymmetry of information (ignorance of consumers of the origin of the product, ignorance of farmers of the real consumer price). High capital investments also mean higher debt pressure and economic dependence towards input suppliers. Areas with more developed commercial production settings were more inclined not to report.

### Non‐monetary costs and benefits of passive surveillance

The present study proposes a methodology for the quantification of incentives and disincentives that cannot be directly valuated in monetary terms, qualified as non‐monetary costs and benefits of the passive surveillance system. It focused on the decision‐making of the most important field actor, the farmer. The relevant disincentives, interpreted as costs of reporting, were the cost of being responsible for the impacts of the release of information (market impacts, mainly at the local level, including animal movement controls) and the transaction costs. One relevant incentive, the benefit of getting information to be used for the implementation of own prevention measures, was also identified and quantified. Farmers did not perceive the actual passive surveillance system as a useful source of information; therefore, the valuation of this benefit was applied separately from that of the expected costs.

A common question in the evaluation of animal health surveillance is the boundaries made between elements entering this strict framework (i.e. the production of information) and those belonging to control or preventive actions (Hasler and Howe, 2012). These elements are actually tightly interconnected. Control actions, being anticipated by actors, are (dis‐)incentives for reporting. Farm disinfection and management of dead birds appear, in the same way as compensation scheme, as an incentive element of the control policy resulting from reporting. It is noteworthy that this incentive was not mentioned in DN. The development of delimited commercial farming area in this region might have reduced the potential conflicts between neighbours about environmental nuisance and risks of poultry disease for public health. Moreover, farmers could easily eliminate their dead animals through feeding domestic crocodiles and pythons bred in this area instead of releasing it in the environment.

This quantification methodology should be further applied to the other types of public actors of the passive surveillance system. Indeed, along the hierarchical chain of veterinary authorities, each level incurs costs and benefits that would deserve to be valued and included in the evaluation of the system. The qualitative results showed that government veterinarians had issues in reporting to upper authorities. Issues in cooperation between local and central veterinary authorities were pointed out by previous evaluations in Vietnam (Fermet‐Quinet et al., 2010).

### Scope and limits of the proposed methodology

The proposed evaluation method may be qualified as a ‘rapid surveillance appraisal’. Previous studies using sociological methods had highlighted the importance of economic and non‐economic factors in the reporting behaviour of animal farmers (Hickler, 2007; Elbers et al., 2010; Fearnley, 2011) and government veterinarians (Sawford et al., 2012; Bronner et al., 2014). However, this study represents the first attempt to develop a comprehensive and replicable methodology for the rapid and systematic identification of costs and benefits directly or indirectly linked to animal disease passive surveillance. It is also the first time, to the authors’ knowledge, that participatory approaches and stated preference methods are used in combination for the purpose of evaluating animal health surveillance systems.

Regarding qualitative investigations, PE tools proved useful in revealing decision‐making factors that are not initially brought forward in interviews. Such factors are difficult to identify. Actors may hide them at first or only unconsciously integrate them in their decision‐making. Questionnaire‐based methods may not be relevant to tackle these factors. Of particular interest were visualization tools used to identify and compare abstract notions such as expectations about positive and negative effects of disease suspicion reporting (‘winners–losers list’) and scoring of factors influencing reporting attitude (PP). These tools were well understood by a majority of participants. The few failed attempts usually were due to a lack of interest or experience of participants in the topic. A special issue in the evaluation lied in the fact that reporting of disease suspicion was considered as an unusual event; therefore, related costs and benefits were in practice mostly or totally avoided. Again, PE approaches helped in tackling the specific needs for this situation. Some of the interviewees were reluctant to share information. This issue was linked with the aim of the study. Underreporting of sanitary events is a sensitive topic, as it is considered a failure to comply with the official regulation. It was particularly true in the study area of LA. The presence of government veterinarians or other government officials during interviews of private actors most likely influenced participants’ responses. It might explain why, in this precise study area, very few poultry farmers mentioned uncertainty in authorities’ reaction and transaction costs as a limit of reporting. It might also explain the numerous mentions of veterinary authorities as sources of information by poultry farmers in this same study area. Nevertheless, in most of the other interviews, participants were not reluctant at addressing sensitive issues such as underreporting, sale of sick animals or distrust in veterinary authorities.

Snowball sampling was a key component of the qualitative assessment (Sadler et al., 2010). It enabled to embrace the diversity of actors impacted by passive surveillance systems and to confront opinions of actors that may not incur the same effects and have different perceptions on the issue. Nevertheless, involvement of private actors with time‐consuming commercial activities (such as medicine sellers, feed sellers and poultry traders) might be difficult and a moderate proportion of refusal is unavoidable with such actors.

This work also constitutes a pilot study for quantification of non‐monetary costs and benefits, combining stated preference methods and PE tools. It demonstrates both the feasibility and the limits of such an approach. Stated preference method is based on the elicitation of specific choices of participants under hypothetic scenarios with specific attributes (Adamowicz et al., 1998; Louviere et al., 2000). The PE approach was well adapted to identify scenarios and relevant attributes that matched participants’ specific perceptions. PP was used in conjoint analysis as a way to capture relative probabilities of decisions in response to change in scenarios attributes. These attributes were progressively adapted by the interviewer all along the exercise until capturing changes in probability that were precisely linked with the factor of interest. This approach significantly differs from classical stated preference methods (Adamowicz and Boxall, 2001). It allowed the greater flexibility that is fundamental to ascertain an understanding and involvement of participants in the exercise. Also, the estimation of prices associated with disease case information provided opportunities to identify factors that were not directly expressed in previous qualitative interviews. Still, the applicability of the tool proved to depend on the context and the ability (or willingness) of participants to consider hypothetic scenarios that could significantly diverge from their personal experience. In some production areas, such as DN, notification of disease cases is too unusual or animal health management is mostly under the responsibility of the private sector. In this case, farmers may perceive the proposed scenarios as too unrealistic to be considered.

The main limitation of this method is a classical limit of qualitative approaches. The need to be applied as in‐depth and flexible investigation allows covering only a limited sample of participants in restricted areas. This limit is more acutely felt in the implementation of the quantification method of non‐monetary costs. The methodology is time‐consuming and flexible between individuals. It cannot be applied on large samples to generate data that may be considered representative of the national level. The method may be considered as a semi‐quantitative tool, and should be regarded as such when integrated in general evaluation frameworks.

Despite these limits, the present study showed on a pilot scale that values of non‐monetary disincentives considered as costs (fear for market impact, transaction cost of reporting) were comparable with values of monetary factors of decision‐making. This result underlines the added value of such semi‐quantitative measurement. Indeed, strictly qualitative investigation does not allow for a comparison of the relative influence of monetary and non‐monetary constraints on poultry farmers’ decision. In general, monetary components were always put forward by participants and non‐monetary components only were revealed after in‐depth interviews.

### Recommendation and perspectives

The study results allowed drawing recommendations for the improvement of HPAI passive surveillance in Vietnam. Firstly, similar rules regarding notification, control and compensation for HPAI or any other suspicion of diseases resulting in sudden and rapid death in poultry (e.g. velogenic Newcastle) should be applied to reduce the uncertainties around the outcomes of official reporting (OIE, 2008). Clear standard rules in terms of compensation should be established yearly and properly communicated along with a simplification of the reporting process and shortening of the delays to get compensations in order to reduce transaction costs associated with reporting.

Secondly, an improvement of passive surveillance performances cannot be dissociated from the improvement of the poultry value chain quality standards (Paul et al., 2013): the need to comply with specific quality standards would discourage the sale of poultry coming from infected farms. The removal of the sick bird specific value chain, if associated with appropriate support of authorities to manage sick and dead poultry, would be likely to encourage poultry farmers to report as the alternative option of selling infected flocks would not be available anymore. Implementation of certification systems of quality and geographical origin associated with improved product traceability and a reduction of the number of intermediates would contribute to decrease the externalities linked to information on disease occurrence incurred by production and upstream sector (Ifft et al., 2010b; Metras et al., 2011).

Lastly, theoretical economic models providing market impact outcomes linked to disease outbreak information have been developed and could be implemented in Vietnam (Sheriff and Osgood, 2010; Saak, 2012). The integration of the outputs of this study within such economic models should be investigated within the context of HPAI passive surveillance in Vietnam to provide valuable information for improvement of disease management (prevention and control) strategy.

## Conclusion

The proposed methodology proved to be quick and efficient in revealing the issues behind the animal disease passive surveillance system, while gaining confidence from all actors involved. The quantification tool showed a clear benefit in terms of communication on the magnitude of disincentives/incentives difficult to appraise. It demonstrated the interest of associating PE tools and econometric methods. Perceived costs and benefits associated with passive surveillance systems are not limited to financial incentives and disincentives. The perceived value of animal disease passive surveillance information was influenced by transaction costs, market impacts, disease management, and usefulness of information for private actors.

## Supporting information


**Figures S1 and S2.** Checklists used in focus group (S1) and individual (S2) interviews of poultry farmers performed in the 2012–2013 survey on the perceived value of the HPAI passive surveillance system in Vietnam.

## References

[zph12212-bib-0001] Adamowicz, V. , and P. Boxall , 2001: Future Directions of Stated Choice Methods for Environment Valuation. University of Alberta, Edmonton, Alberta, Canada.

[zph12212-bib-0002] Adamowicz, W. , P. Boxall , M. Williams , and J. Louviere , 1998: Stated preference approaches for measuring passive use values: choice experiments and contingent valuation. Am. J. Agr. Econ. 80, 64–75.

[zph12212-bib-0003] Agrifood Consulting International , 2006: Poultry Sector Rehabilitation Project – Phase I: The Impact of Avian Influenza on Poultry Sector Restructuring and its Socio‐Economic Effects. Prepared for the Food and Agriculture Organization of the United Nations. Agrifood Consulting International, Bethesda, MD.

[zph12212-bib-0004] Bonfoh, B. , K. Schwabenbauer , D. Wallinga , J. Hartung , E. Schelling , J. Zinsstag , F.‐X. Meslin , R. Tschopp , J. A. Akakpo , and M. Tanner , 2010: Human health hazards associated with livestock production In: Steinfeld H., Mooney H. A., Schneider F., and Neveille L. E. (eds), Livestock in a Changing Landscape: Drivers, Consequences and Responses, pp. 197–219. Island Press, Washington, DC.

[zph12212-bib-0005] Bowen, G. A. , 2008: Naturalistic inquiry and the saturation concept: a research note. Qual. Res. 8, 137–152.

[zph12212-bib-0006] Bronner, A. , V. Henaux , N. Fortane , P. Hendrikx , and D. Calavas , 2014: Why do farmers and veterinarians not report all bovine abortions, as requested by the clinical brucellosis surveillance system in France? BMC Vet. Res. 10, 93. 24762103 10.1186/1746-6148-10-93 PMC4036594

[zph12212-bib-0007] Catley, A. , R. G. Alders , and J. L. Wood , 2012: Participatory epidemiology: approaches, methods, experiences. Vet. J. 191, 151–160. 21856195 10.1016/j.tvjl.2011.03.010

[zph12212-bib-0008] Chilonda, P. , and G. Van Huylenbroeck , 2001: A conceptual framework for the economic analysis of factors influencing decision‐making of small‐scale farmers in animal health management. Rev. Sci. Tech. 20, 687–700. 11732411 10.20506/rst.20.3.1302

[zph12212-bib-0009] Croissant, Y. , 2013: mlogit: Multinomial Logit Model. R package version 0.2‐4. Available at: http://CRAN.R-project.org/package=mlogit (accessed on 30 November 2014).

[zph12212-bib-0010] Davies, S. , 2012: Nowhere to hide: informal disease surveillance networks tracing state behaviour. Global Change Peace Secur. 24, 95–107.

[zph12212-bib-0011] Department of Animal Health , 2011: Official Guide of avian influenza surveillance in years 2011‐2012. 1109/TY‐DT. Department of Animal Health of Vietnam, Hanoi, Vietnam.

[zph12212-bib-0012] Department of Animal Health , 2014: List of Notified H5N1 HPAI Outbreaks. Department of Animal Health of Vietnam, Hanoi, Vietnam.

[zph12212-bib-0013] Desvaux, S. , and M. Figuie , 2011: Formal and informal surveillance systems. How to build bridges? Bulletin de l'AEEMA 59–60, 352–355.

[zph12212-bib-0014] Diao, X. , 2009: Economywide Impact of Avian Flu in Ghana. A Dynamic CGE Model Analysis. International Food Policy Research Institute, Washington, DC.

[zph12212-bib-0015] Drewe, J. A. , L. J. Hoinville , A. J. Cook , T. Floyd , and K. D. Stark , 2011: Evaluation of animal and public health surveillance systems: a systematic review. Epidemiol. Infect. 140, 575–590. doi:10.1017/S0950268811002160. 22074638 10.1017/S0950268811002160

[zph12212-bib-0016] Elbers, A. R. , M. J. Gorgievski‐Duijvesteijn , K. Zarafshani , and G. Koch , 2010: To report or not to report: a psychosocial investigation aimed at improving early detection of avian influenza outbreaks. Rev. Sci. Tech. 29, 435–449. 21309445 10.20506/rst.29.3.1988

[zph12212-bib-0017] FAO , 2011a: Approaches to Controlling, Preventing and Eliminating H5N1 Highly Pathogenic Avian Influenza in Endemic Countries. Food and Agriculture Organization of the United Nations, Rome.

[zph12212-bib-0018] FAO , 2011b: Challenges of Animal Health Surveillance systems and Surveillance for Animal Diseases and Zoonoses. Food and Agriculture Organisation of the United Nations, Rome.

[zph12212-bib-0019] Fearnley, L. , 2011: Disputing Efficacy: Poultry Farmers and Pharmaceutical Exchange in Nanchang County. Jiangxi, Beijing, China.

[zph12212-bib-0020] Fermet‐Quinet, E. , M. Edan , and J. Stratton , 2010: Tool for the Evaluation of Performance of Veterinary Services – Vietnam. OIE ‐ World Organisation of Animal Health, Paris.

[zph12212-bib-0021] Figuie, M. , and T. Fournier , 2008: Avian influenza in Vietnam: chicken‐hearted consumers? Risk Anal. 28, 441–451. 18419660 10.1111/j.1539-6924.2008.01039.x

[zph12212-bib-0022] Figuie, M. , A. T. Pham , and P. Moustier , 2013: Avian flu in the food chain. The reshape of the agro‐industrial sector in Vietnam. Revue d'Etudes en Agriculture en Environnement 94, 397–419.

[zph12212-bib-0023] Fournie, G. , J. Guitian , S. Desvaux , P. Mangtani , S. Ly , C. C. Vu , S. San , H. D. Do , D. Holl , D. U. Pfeiffer , S. Vong , and A. C. Ghani , 2012: Identifying live bird markets with the potential to act as reservoirs of avian influenza A (H5N1) virus: a survey in Northern Viet Nam and Cambodia. PLoS One 6, e37986. 22675502 10.1371/journal.pone.0037986 PMC3366999

[zph12212-bib-0024] General Statistics Office of Vietnam , 2012: Results of the 2011 Rural, Agriculture and Fishery Census. Statistical Publishing House, Hanoi, Vietnam.

[zph12212-bib-0025] Graneheim, U. H. , and B. Lundman , 2004: Qualitative content analysis in nursing research: concepts, procedures and measures to achieve trustworthiness. Nurse Educ. Today 24, 105–112. 14769454 10.1016/j.nedt.2003.10.001

[zph12212-bib-0026] Hasler, B. , and K. Howe , 2012: Evaluating the role of surveillance in national policies for animal health. EuroChoices 11, 39–44.

[zph12212-bib-0027] Hickler, B. , 2007: Bridging the Gap between HPAI “Awareness” and Practice in Cambodia. Recommendations from an Anthropological Participatory Assessment. Food and Agriculture Organisation of the United Nations, Rome.

[zph12212-bib-0028] Hoinville, L. , 2011: Animal Health Surveillance Terminology. Final Report from Pre‐ICAHS Workshop. Available at: http://www.fp7-risksur.eu/sites/fp7-risksur.eu/files/partner_logos/icahs-workshop-2011_surveillance_tewrminology_report_V1.2.pdf (accessed on 15 December 2014).

[zph12212-bib-0029] Hong Hanh, P. T. , S. Burgos , and D. Roland‐Holst , 2007: The Poultry Sector in Viet Nam: Prospects for Smallholder Producers in the Aftermath of the HPAI Crisis. Pro‐Poor Livestock Policy Initiative Research Report, Food and Agriculture Organisation of the United Nations, Rome.

[zph12212-bib-0030] Hopp, P. , S. Vatn , and J. Jarp , 2007: Norwegian farmers’ vigilance in reporting sheep showing scrapie‐associated signs. BMC Vet. Res. 3, 34. 18076757 10.1186/1746-6148-3-34 PMC2246117

[zph12212-bib-0031] Ifft, J. , D. A. T. Nguyen , D. L. Nguyen , J. Otte , and D. Roland‐Holst , 2010a: Poultry Demand in Ha Noi and Ho Chi Minh City. HPAI Research Brief No. 24. Controlling Avian Flu and Protecting People's Livelihoods DFID, FAO, RDRC and RVC, London.

[zph12212-bib-0032] Ifft, J. , D. A. T. Nguyen , D. L. Nguyen , J. Otte , and D. Roland‐Holst , 2010b: Safety Certified Free‐Range Duck Supply Chains Enhance both Public Health and Livelihoods. HPAI Research Brief No. 23. Controlling Avian Flu and Protecting People's Livelihoods. DFID, FAO, RDRC and RVC, London.

[zph12212-bib-0033] Legendre, P. , 2005: Species associations: the Kendall coefficient of concordance revisited. J. Agric. Biol. Environ. Stat. 10, 226–245.

[zph12212-bib-0034] Louviere, J. J. , D. A. Hensher , and J. D. Swait , 2000: Stated Choice Methods: Analysis and Applications, pp. 418 Cambridge University Press, Cambridge.

[zph12212-bib-0035] Mariner, J. C. , and R. Paskin , 2000: Manual on Participatory Epidemiology. Methods for the Collection of Action‐Orientated Epidemiological Intelligence. Food and Agriculture Organisation of the United Nations, Rome.

[zph12212-bib-0036] Metras, R. , R. J. Magalhaes , Q. H. Dinh , G. Fournie , J. Gilbert , D. D. Huu , D. Roland‐Holst , J. Otte , and D. U. Pfeiffer , 2011: An assessment of the feasibility of a poultry tracing scheme for smallholders in Vietnam. Rev. Sci. Tech. 30, 703–714. 22435183 10.20506/rst.30.3.2072

[zph12212-bib-0037] Minh, P. Q. , R. S. Morris , B. Schauer , M. Stevenson , J. Benschop , H. V. Nam , and R. Jackson , 2009: Spatio‐temporal epidemiology of highly pathogenic avian influenza outbreaks in the two deltas of Vietnam during 2003‐2007. Prev. Vet. Med. 89, 16–24. 19232765 10.1016/j.prevetmed.2009.01.004

[zph12212-bib-0038] Minh, P. Q. , M. A. Stevenson , C. Jewell , N. French , and B. Schauer , 2011: Spatio–temporal analyses of highly pathogenic avian influenza H5N1 outbreaks in the Mekong River Delta, Vietnam, 2009. Spat. Spatiotemporal. Epidemiol. 2, 49–57. 22749548 10.1016/j.sste.2010.11.001

[zph12212-bib-0039] NSCAI , 2012: Report on Prevention and Control of Avian Influenza. National Steering Committee for Avian Influenza, Hanoi, Vietnam.

[zph12212-bib-0040] OIE , 2008: OIE Technical Disease Cards. Highly Pathogenic Avian Influenza.

[zph12212-bib-0041] Oksanen, J. , F. G. Blanchet , R. Kindt , P. Legendre , P. R. Minchin , R. B. O'Hara , G. L. Simpson , P. Solymos , M. H. H. Stevens , and H. Wagner , 2014: vegan: Community Ecology Package. Available at: http://CRAN.R-project.org/package=vegan (accessed on 17 November 2014).

[zph12212-bib-0042] Otte, J. , D. Roland‐Holst , and D. Pfeiffer , 2006: HPAI Control Measures and Household Incomes in Viet Nam. FAO Pro‐Poor Livestock Policy Initiative. Food and Agriculture Organisation of the United Nations, Rome.

[zph12212-bib-0043] Paul, M. , V. Baritaux , S. Wongnarkpet , C. Poolkhet , W. Thanapongtharm , F. Roger , P. Bonnet , and C. Ducrot , 2013: Practices associated with Highly Pathogenic Avian Influenza spread in traditional poultry marketing chains: social and economic perspectives. Acta Trop. 126, 43–53. 23337390 10.1016/j.actatropica.2013.01.008

[zph12212-bib-0044] Pfeiffer, D. U. , P. Q. Minh , V. Martin , M. Epprecht , and M. J. Otte , 2007: An analysis of the spatial and temporal patterns of highly pathogenic avian influenza occurrence in Vietnam using national surveillance data. Vet. J. 174, 302–309. 17604193 10.1016/j.tvjl.2007.05.010

[zph12212-bib-0045] R Core Team , 2014: R: A Language and Environment for Statistical Computing. R Foundation for Statistical Computing, Vienna, Austria.

[zph12212-bib-0046] Rodriguez, U.‐P. E. , Y. T. Garcia , A. G. Garcia , and R. L. Tan , 2007: Can trade policies soften the economic impacts of an avian influenza outbreak? Simulations from a CGE Model of the Philippines. Asian J. Agric. Dev. 4, 41–50.

[zph12212-bib-0047] Roland‐Holst, D. , M. Epprecht , and J. Otte , 2007: External Shocks, Producer Risk, and Adjustment in Smallholder Livestock Production: The Case of HPAI in Viet Nam. University of California, Berkeley, CA.

[zph12212-bib-0048] Saak, A. E. , 2012: Infectious Disease Detection with Private Information. IFPRI Discussion Paper. International Food Policy Research Institute, Washington, DC.

[zph12212-bib-0049] Sadler, G. R. , H. C. Lee , R. S. Lim , and J. Fullerton , 2010: Recruitment of hard‐to‐reach population subgroups via adaptations of the snowball sampling strategy. Nurs. Health. Sci. 12, 369–374. 20727089 10.1111/j.1442-2018.2010.00541.x PMC3222300

[zph12212-bib-0050] Sawford, K. , A. R. Vollman , and C. Stephen , 2012: A focused ethnographic study of Sri Lankan government field veterinarians’ decision making about diagnostic laboratory submissions and perceptions of surveillance. PLoS One 7, e48035. 23133542 10.1371/journal.pone.0048035 PMC3485039

[zph12212-bib-0051] Scott, A. E. , K. W. Forsythe , and C. L. Johnson , 2012: National animal health surveillance: return on investment. Prev. Vet. Med. 105, 265–270. 22305661 10.1016/j.prevetmed.2012.01.007

[zph12212-bib-0052] Sheriff, G. , and D. Osgood , 2010: Disease forecasts and livestock health disclosure: a shepherd's dilemma. Am. J. Agric. Econ. 92, 776–788.

[zph12212-bib-0053] Soares Magalhaes, R. , H. D. Quoc , and L. T. Kim Lan , 2007: Farm Gate Trade Patterns and Trade at Live Poultry Markets Supplying Ha Noi: Results of a Rapid Rural Appraisal. Royal Veterinary College, London.

[zph12212-bib-0054] Thurlow, J. , 2011: Consequences of avian flu for growth and poverty: A CGE analysis for Kenya. Afr. Dev. Rev. 23, 276–288.

[zph12212-bib-0055] Tung, D. X. , and A. Costales , 2007: Market Participation of Smallholder Poultry Producers in Northern Viet Nam. Food and Agriculture Organisation of the United Nations, Rome.

[zph12212-bib-0056] Vergne, T. , V. Grosbois , B. Durand , F. Goutard , C. Bellet , D. Holl , F. Roger , and B. Dufour , 2012: A capture‐recapture analysis in a challenging environment: assessing the epidemiological situation of foot‐and‐mouth disease in Cambodia. Prev. Vet. Med. 105, 235–243. 22225773 10.1016/j.prevetmed.2011.12.008

