# Supplementary material for: The Perceived Value of Passive Animal Health Surveillance: The Case of Highly Pathogenic Avian Influenza in Vietnam
Source: Zoonoses Public Health. 2015 Jul 3;63(2):112–28. doi: 10.1111/zph.12212 (PMC4758386; doi:10.1111/zph.12212)
Supplement: Supplementary file 1 — Figures S1 and S2. Checklists used in focus group (S1) and individual (S2) interviews of poultry farmers performed in the 2012–2013 survey on the perceived value of the HPAI passive surveillance system in Vietnam. [file ZPH-63-112-s001.pdf]

Checklist

Semi-structured interview

factors influencing poultry farmers' decision-making

1. **Understand the motivations of the interviewee to disclose or hide information**

    1.1. *Make the interviewee in the situation of a choice to notify or not:*

First: Checking of the history of the farm regarding the case definition adopted:

Case definition: more than 50% of mortality in 5 days.

⇨ *If this situation happened in the interviewee's farm:* reasons farmers decided to share the information with some actors + the reasons he decided to report the disease to authorities or not

⇨ *If the situation did not happen:* imagine it happens in his farm. Reasons to report to veterinary authorities or not.

    1.2. *Explore more in details the factors of decision:*

⇨ List the consequences of the report of disease case

⇨ List the consequences for a positive case and declaration of a notifiable disease

⇨ List the consequences if the disease case is not reported

2. **Precise identification of the actors influencing the participants' decision to report and the motivations behind these influences**

*TOOL = list of actors (winner-loser list)*

Among the listed persons identification of the ones that can influence the decision of the interviewee to report or not report.

3. **Semi-quantification of the importance of the different reasons to report or not report**

*TOOL = Proportional piling => ascribe a weight to the different arguments to report or not report.*

4. **Sources of information**

⇨ List sources of information of the interviewee on above-mentioned case definition.

⇨ Reasons of the interest of these sources of information
