# Supplementary material for: The Perceived Value of Passive Animal Health Surveillance: The Case of Highly Pathogenic Avian Influenza in Vietnam
Source: Zoonoses Public Health. 2015 Jul 3;63(2):112–28. doi: 10.1111/zph.12212 (PMC4758386; doi:10.1111/zph.12212)
Supplement: Supplementary file 2 [file ZPH-63-112-s002.pdf]

Checklist

Focus groups poultry farmers

1. **Description of poultry farming systems and associated value chain**
    - Sources of chicks
    - Sources of feed
    - Sources of medicines
    - Sale of farm products

2. **Description of problems met in poultry farming :**

TOOL: simple ranking

3. **Description of animal diseases impacting poultry farms**

TOOL: PP

- Names given to sanitary events
- Impact on income
- Rate of mortality and duration of disease

4. **Description of management of diseases matching the case definition**

Case definition: more than 50% of mortality in 5 days.

TOOL: PP

- Actors contacted and actions taken
